# Evolutionary Steps in the Analytics of Primordial Metabolic Evolution †

**DOI:** 10.3390/life9020050

**Published:** 2019-06-18

**Authors:** Thomas Geisberger, Philippe Diederich, Thomas Steiner, Wolfgang Eisenreich, Philippe Schmitt-Kopplin, Claudia Huber

**Affiliations:** 1 Department of Biochemistry, Technical University of Munich, 80333 Munich, Germany; thomas.geisberger@tum.de (T.G.); thomas.steiner@tum.de (T.S.); wolfgang.eisenreich@mytum.de (W.E.); 2 Research Unit Analytical BioGeoChemistry, Helmholtz Zentrum München–German Research Center for Environmental Health, 85764 Neuherberg, Germany; philippe.diederich@helmholtz-muenchen.de (P.D.); schmitt-kopplin@helmholtz-muenchen.de (P.S.-K.)

**Keywords:** origin of life, GC-MS, LC-MS, NMR, FT-ICR, stable isotopes, reaction network

## Abstract

Experimental studies of primordial metabolic evolution are based on multi-component reactions which typically result in highly complex product mixtures. The detection and structural assignment of these products crucially depends on sensitive and selective analytical procedures. Progress in the instrumentation of these methods steadily lowered the detection limits to concentrations in the pico molar range. At the same time, conceptual improvements in chromatography, nuclear magnetic resonance (NMR) and mass spectrometry dramatically increased the resolution power as well as throughput, now, allowing the simultaneous detection and structural determination of hundreds to thousands of compounds in complex mixtures. In retrospective, the development of these analytical methods occurred stepwise in a kind of evolutionary process that is reminiscent of steps occurring in the evolution of metabolism under chemoautotrophic conditions. This can be nicely exemplified in the analytical procedures used in our own studies that are based on Wächtershäuser’s theory for metabolic evolution under Fe/Ni-catalyzed volcanic aqueous conditions. At the onset of these studies, gas chromatography (GC) and GC-MS (mass spectrometry) was optimized to detect specific low molecular weight products (<200 Da) in a targeted approach, e.g., methyl thioacetate, amino acids, hydroxy acids, and closely related molecules. Liquid chromatography mass spectrometry (LC-MS) was utilized for the detection of larger molecules including peptides exceeding a molecular weight of 200 Da. Although being less sensitive than GC-MS or LC-MS, NMR spectroscopy benefitted the structural determination of relevant products, such as intermediates involved in a putative primordial peptide cycle. In future, Fourier transform ion cyclotron resonance mass spectrometry (FT-ICR-MS) seems to develop as a complementary method to analyze the compositional space of the products and reaction clusters in a non-targeted approach at unprecedented sensitivity and mass resolution (700,000 for m/z 250). Stable isotope labeling was important to differentiate between reaction products and artifacts but also to reveal the mechanisms of product formation. In this review; we summarize some of the developmental steps and key improvements in analytical procedures mainly used in own studies of metabolic evolution.

## 1. Introduction

In experiments simulating conditions for a potential chemoautotrophic origin of metabolism, simple inorganic C_1_- or C_2_-educts are used as precursors for the formation of organic molecules that could also have established the first “metabolic” reaction networks in the Universe [1,2]. The product mixtures obtained under these conditions are characterized by hundreds to thousands of compounds, most of them being at very low concentrations. This constraint is valid for many scenarios, which may be as different in their conceptual background as molecules formed on Earth under putative Hadean conditions or molecules formed in space and detected as product mixtures obtained from meteorites. It is immediately obvious that the detection and structural assignment of these products is highly demanding. In the history of “origin-of-life” research, the analytical methods have made extensive progress. At the beginning of this research, methods were available that allowed the separation and chemical detection of only a few compounds from the reaction mixtures via a limited set of chromatographic methods, which was followed by additional identification via mass spectrometry (MS) and nuclear magnetic resonance spectrometry (NMR) mainly in targeted approaches. Modern methods now allow for the simultaneous detection and characterization of thousands of compounds in non-targeted approaches. The purpose of this review is to show the evolution of these analytical techniques and their impacts on the research about the “origin-of-life”.

Our own focus in “origin-of-life” research is based on Wächtershäuser’s theory of a putative chemoautotrophic origin of metabolism catalyzed by transition metal sulfides under conditions of volcanic hydrothermal vents [3,4,5]. However, the challenge of analyzing low concentrated product mixtures containing a high number of compounds is also valid for other experimental studies in the field of “origin-of-life”. In the history of these studies, the pioneering Miller–Urey experiment [6] was one of the most popular ones. Through electric discharges CH_4_, NH_3_, H_2_O, and H_2_ were reacted to amino acids like glycine, alanine, amino butyrate, and aspartate, as shown by paper chromatography (PC) in the form of their ninhydrin adducts. Using this method, already introduced in 1944 by Consden et al. [7], an artificial mixture of 22 amino acids could already be identified in a single experiment at amounts of 0.2–0.4 mg for each amino acid. Despite its simplicity, the method allowed for detection in the low micro molar range [8,9]. Later, the method was replaced by thin layer chromatography (TLC) which offered a better handling than PC [10]. For quite a long period of “origin-of-life” research, amino acids and nucleobases like adenine were identified by TLC [11]. However, as a bottleneck of this method, the number of compounds that can be simultaneously identified is rather low [12] and both PC and TLC relied on the comparison with retention indices of reference standards and were therefore error prone. Some of these problems could be addressed by two-dimensional TLC, but more sophisticated techniques were necessary to characterize more products in these reaction mixtures.

In an important developmental step, gas chromatography (GC) was introduced that overcame many of the disadvantages in PC or TLC. Already in the 1940s and 1950s, GC was used for the separation of CO_2_ from air [13,14,15] and for the separation of fatty acids [16]. During the early years of GC development, mainly packed columns were used which were limited to a length of about 5 m. Through the invention of fused silica columns in the 1980s, separation, sensitivity, and reproducibility were dramatically increased and since then, GC is a standard procedure in almost any field of analytical chemistry [17]. Detection limits are in the low micro molar to pico molar range, depending on substance classes and eventually on derivatization efficiency [18]. In typical settings, up to several hundreds of volatile and thermally stable compounds can be separated in a single GC run within 30–60 min. GC is combined with different detectors, namely the flame ionization detector (FID) [19,20] which offers a high linearity over 7 orders of magnitude and a high sensitivity in the pg range. Today, FIDs are complemented with mass-sensitive detectors [21]. Here, a quadrupole-based detector with electron impact offers the most stable and low-maintenance handling for multiple usages. In typical settings, four parallel rods allow a mass selection dependent on the m/z values at reasonable resolution (0.5–0.1 au). Electron impact ionization leads to a characteristic fragmentation pattern of the original molecule which can be used for product-identification. Product identification is supported by standard libraries with hundreds of thousands stored mass spectra. Notably, GC-MS does not necessarily require a corresponding reference sample, in sharp contrast to the earlier PC, TLC, and GC-FID methods. The basic requirement for GC-MS analysis, namely the volatility and thermal stability of the analytes did not change over the years of application. This problem can be overcome, in part, by chemical derivatization prior to GC-MS analysis, for example by the introduction of appropriate functions e.g., of alkyl silyl groups into the products under study such as amino acids, hydroxyl acids, or related compounds.

However, to cover the full chemical space of product formation and to avoid error-prone chemical derivatization, complementary analytical methods were necessary. High pressure liquid chromatography (HPLC) coupled to UV/VIS detectors are well established as a means for the separation of non-volatile compounds through small particle columns since about the 1960s [22,23]. The coupling to MS detectors was an unsolved problem for a long time, as liquids produce high amounts of vapor and electron ionization has to be performed under high vacuum. Only in 1973, [24] a method was introduced where the liquid eluate could be sprayed into a chemical ionization source. In 1978, a liquid chromatography mass spectrometry (LC-MS) interface (called thermospray) was reported [25], which now allowed the combination of reverse phase chromatography with MS detection. LC-MS nowadays offers highly sensitive detection of a large number of analytes which are at least comparable or even superior to GC-MS and it is no surprise that LC-MS is now also among the key methods for “origin-of-life” experiments. As a general constraint, however, LC-MS is higher priced and less robust than GC-MS, in particular for routine usage.

Another highly priced analytical method in the field is nuclear magnetic resonance spectroscopy (NMR). Nowadays, the usage of high-field magnets (up to 1.2 GHz ^1^H NMR frequency), sensitive cryo-probes and sophisticated multi-dimensional experiments provide quite unbiased information about low molecular weight metabolites in complex mixtures (e.g., extracts or body fluids). On the basis of high-resolved unique resonance frequencies and coupling patterns of molecules in solution, up to about 100 different compounds can be identified in these fluids. Quite obviously, the technique is also powerful in detecting reaction products from complex synthetic mixtures, such as compound cocktails obtained from “origin-of-life” experiments. Nevertheless, NMR is still rarely used in these studies, mostly due to its low intrinsic sensitivity in comparison to MS techniques.

Quite recently, FT-ICR-MS has been added as a high-resolution mass spectrometric method that allows the discrimination of substances differing in a mass of only half an electron (at m/z 500). Sub-ppm mass accuracy in conjunction with ultrahigh mass resolution allows for the direct annotation of the elemental composition for the detected ions over a high mass range up to m/z 700 (m/z 1000 in interpolations) [26]. This annotation makes the characterization of even highly complex substance mixtures possible. Complex mixtures are readily found in nature, for example terrestrial natural organic matter (NOM) and extraterrestrial NOM extracted from meteorites, where analysis by FT-ICR-MS has already proven its usefulness [27,28].

**Figure 1:**
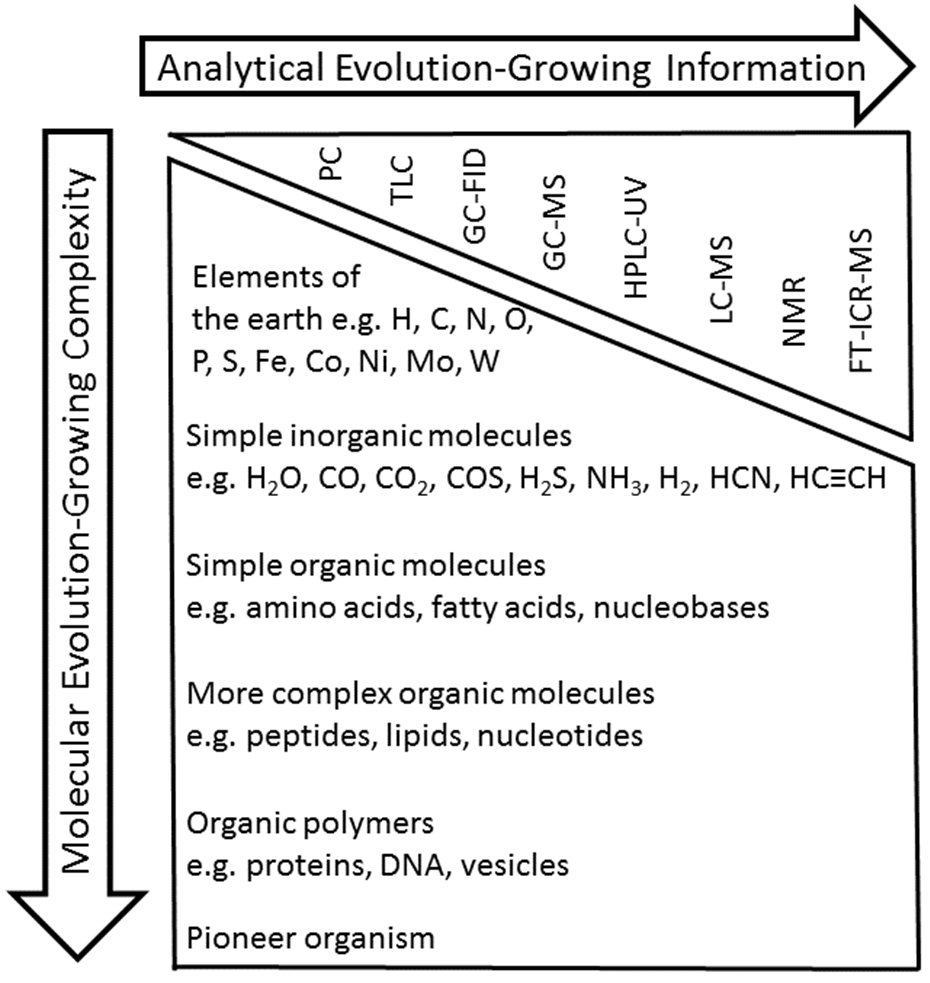
Evolution of the molecular complexity in the “origin-of-life” scenario triggered by a chemoautotrophic evolution of metabolism and evolution of methods for its analysis. Catalyzed by minerals present in the crust of early Earth, simple organic molecules were formed from simple inorganic molecules. Through polymerization biomolecules could have emerged which allowed the evolution of cells and the “Pioneer organism”. This evolution of molecular complexity is reflected by the evolution of analytical tools and the amount of information delivered by them. PC: paper chromatography; TLC: thin layer chromatography; GC-FID: gas chromatography-flame ionization detector; HPLC-UV: high pressure liquid chromatography-UV; GC-MS: gas chromatography mass spectrometry; LC-MS: liquid chromatography mass spectrometry; NMR: nuclear magnetic resonance; FT-ICR-MS: Fourier transform ion cyclotron resonance mass spectrometry.

In the following, we exemplify some of the analytical advances during the last 25 years by our own work on metabolic evolution following Wächtershäuser’s theory for a chemoautotrophic “origin-of-life” [2,5]. Inorganic elements of the early Earth such as transition metal sulfides, containing iron or nickel, catalyze reactions of simple inorganic volatile compounds present in volcanic gases. For example, carbon fixation from CO, CO_2_, and HCN afforded simple organic molecules including amino acids, hydroxy acids, fatty acids, and nucleobases. Through further surface catalyzed reactions, more and more complex organic molecules like peptides, lipids and nucleotides have been formed followed by polymerization to vesicles, proteins, RNA, and DNA. These evolutionary steps culminated in the formation of the so called “Pioneer organism” [5]. To determine the growing complexity of this metabolic evolution, research of the “origin-of-life” benefitted from the evolution of analytical methods (at a different time scale) with ever more sophisticated procedures (Figure 1).

## 2. GC-FID and GC-MS Analysis as Robust Tools for Detecting Simple Organic Molecules Formed Under Primordial Conditions

In typical settings of our research on the chemoautotrophic origin of metabolism, we simulate in laboratory experiments volcanic hydrothermal vent conditions and transition metal sulfide catalysis. Reactions are performed in serum flasks or small laboratory pressure vessels and product formation is screened by a variety of analytical methods which developed over the last decades.

In our early experiments (1997), we modeled reactions of the reductive acetyl–CoA pathway under these primordial reaction settings. This pathway is considered as an ancient carbon fixation pathway, which generates acetyl-CoA from CO_2_ and/or CO. Notably, the key enzyme, acetyl-CoA synthase, contains an iron–nickel–sulfur center [29,30,31,32]. The acetyl moiety is formed from CO and a methyl group, which is transferred from *N*-methyl pterin to the Ni reaction center [29]. Instead of *N*-methyl pterin, we used methyl thiol, a constituent of volcanic gases [33] and a reaction product from CO_2_ and H_2_S in the presence of FeS [34]. In combination with CO, we found that an aqueous slurry of freshly precipitated NiS and FeS yields the activated thioester CH_3_-CO-SCH_3_, which hydrolyzes to acetic acid within the aqueous reaction conditions. All of these reaction settings can be presumed to be relevant in a volcanic hydrothermal scenario. Indeed, iron and nickel compounds mainly as sulfides are ubiquitous and coexisting minerals at such sites [35,36].

**Figure 2:**
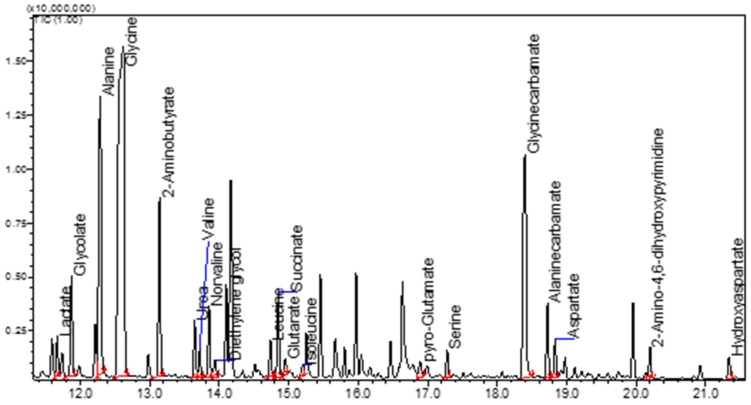
GC-MS trace with identified products from an experiment performed at 160 °C at 75 bar for 20 h with Ni, CO, and CN [38].

**Figure 3:**
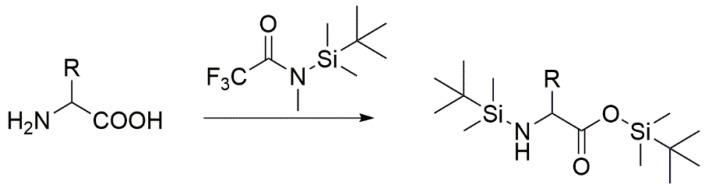
Formation of amino acid MTBSTFA derivatives used for GC-MS analysis.

Acetic acid and its thioester were volatile enough to be analyzed without derivatization. Acetic acid was analyzed directly from the acidified aqueous solution on a packed neopentyl glycol succinate-GC-column (NPGS) with FID detection, whereas the thioester was extracted with hexane and identified through GC-MS [37]. During the next 10 years, the reaction setting was extended by the addition of KCN (soluble ferro cyanides were found in thermal springs [38]), mixed Ni^2+^ and Fe^2+^ hydroxides [35] and by higher pressures (up to 75 bar) and temperatures (80–120 °C). In these mixtures, we could then identify by GC-MS (as MTBSTFA derivatives) a cocktail of α-amino acids, α-keto acids, and α-hydroxy acids such as glycine, alanine, serine, glycolate, lactate, glycerate, α-amino- and hydroxy-valerate (Figure 2) [39]. This derivatization method replaces all acidic protons with *tert*-butyldimethylsilyl residues (Figure 3) which are volatile and more stable than simple trimethylsilyl derivatives and which also show a more specific fragmentation pattern [40].

Reactions performed with K^13^CN proofed a mixed usage of cyano and non-cyano carbons for the formation of the carbon skeleton. This mixed use of cyano, CO, or methylthio ligands opens evolutionary possibilities depending on the present conditions. The additional use of ^15^NH_4_Cl showed incorporation of the ^15^N label into the products containing an amino group. For the formation of amino acids, hydroxy acids, and the corresponding amides, the temperature optimum at about 160–180 °C was determined [41,42]. Incorporation of ^13^C or ^15^N into products can be easily observed through mass selective analytical tools like GC-MS by the corresponding shift of the observed masses. The example shows that stable-isotope labeling is a key method to elucidate the mechanisms of product formation in considerable detail.

Inspired by the extant formation of acetaldehyde through acetylene dehydratase, a tungsten-iron–nickel enzyme [43], we used acetylene, present in fumarolic gases [44] as an additional putative carbon precursor (in combination with CO) in our settings. Indeed, short chain C_2_–C_9_ fatty acids could be detected in these reaction mixtures using GC-MS procedure described above [45]. For safety reasons, these reactions had to be performed at lower pressures (1–2 bar). Assuming a much higher pressure under deep sea conditions, the formation of longer fatty acids with the potential for vesicle formation may be postulated.

There is now a considerable number of further studies on “origin-of-life” scenarios using GC-FID or GC-MS as analytical tools. From meteorites and chondrites, potential precursor molecules in the evolution of metabolism could be detected by GC-MS and GC-FID [46,47]. In the early 1990s, amino acids were also analyzed by Russell and coworkers using GC-MS [48]. In 2000, Cody et al. [49] performed experiments similar to our ones using transition metal sulfides as catalysts and analysis was again done by GC-MS. Moran and coworkers also worked on universal precursors and transition metal catalysis in “origin-of-life” setups using GC-MS as detection method [50,51,52]. Quite recently, Pallmann et al. [53] studied the effect of synthetic analogues to Schreibersite (an iron-nickel phosphide mineral) acting as catalyst for the formose reaction. The identification was done amongst other techniques by GC-MS. Moreover the occurrence of amino acids as well as ketones and aldehydes on meteorites, for example the Murchison or Murray meteorite, could also be shown with the help of GC-MS [54,55,56,57,58,59].

## 3. HPLC-UV and LC-MS for the Elucidation of the Formation of More Complex Molecules Like Peptides

**Figure 4:**
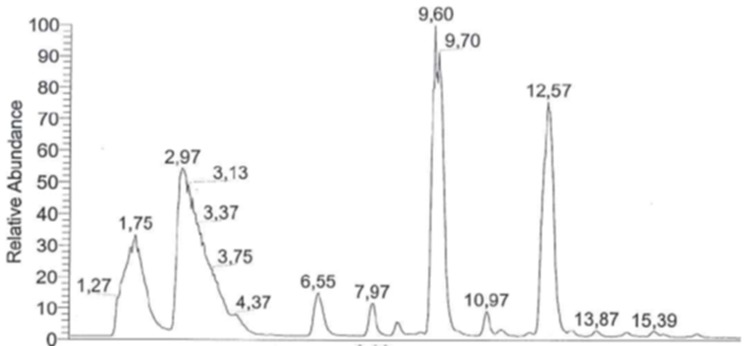
LC-MS trace showing the TIC (total ion current) of a peptide forming experiment starting from phenylalanine [69]. Retention times are as follows: Phe: 1.75 min; PhePhe: 2.97 and 6.55 min; PhePhePhe: 7.97 and 10.97 min; PhePhe +44au: 9.6 and 11.1 min; PhePhe +26au: 12.57 PhePhePhe +44au: 13.87 min; PhePhePhe +26au: 15.39 min.

The formation of peptides under primordial FeS-NiS conditions constitutes one of the great challenges in the “origin-of-life” research. Under aqueous conditions, a condensation reaction such as the formation of a peptide bond is strongly unfavorable. In synthetic chemistry, this problem is solved by the usage of α-amino acid *N*-carboxyanhydrides (NCAs) as activated forms of amino acids prepared for the peptide formation [60]. In 1998, we found that under our typical primordial conditions amino acids can be converted into peptides. More specifically, glycine, phenylalanine and tyrosine were reacted in an aqueous slurry of FeS and NiS with CO in the presence of H_2_S or CH_3_SH at 100 °C. Alternatively, COS was used as activating gas. Due to the low volatility and low yields of derivatization, GC-MS could not be used for a sensitive monitoring of peptides. Therefore, we introduced LC-MS to identify di-, tri-, and tetra-peptides formed after 1–4 days under these conditions. For separation, a reversed phase column (RP-C18) was used with a water-acetonitrile gradient for product elution (Figure 4). Mass detection was here done after positive ESI ionization in a mass range of 100–2000 au. UV detection at 254 nm was done simultaneously [61]. All peptides were formed as racemic mixtures even when the starting amino acid was enantiomerically pure. Facile peptide formation with the help of COS was later investigated in more detail by Leman et al. [62].

LC is well suited for peptide analyses comprising 2 to about 20 amino acids. Nevertheless, the “chemical space” is much broader in “origin-of-life” research and only a few examples are mentioned in the following, where LC-based methods were used as the analytical tool. Kimoto and Fujinaga observed organic polymers containing amino acids, which were analyzed by HPLC on a RP-C18 column [63,64]. Another work based on LC-MS (RP-C8 column) demonstrated formation of fructose-1,6-bisphosphate in ice from the precursors, glyceraldehyde 3-phosphate, and dihydroxyacetone phosphate [65]. Keller and Ralser established a method for LC-MRM (multiple reaction monitoring) to measure Fe^2+^-based non-enzymatic glycolysis and pentose phosphate pathways [66] and the influence of sulfate radicals on the formation and reactions of TCA-intermediates [67]. Fahrenbach et al. also used LC-MS to follow the reaction of 2-aminooxazole and 2-aminoimidazole with glyceraldehyde to form precursors of nucleotides [68].

## 4. NMR as a Powerful Tool to Determine the Structures of Complex Unknown Intermediates

**Figure 5:**
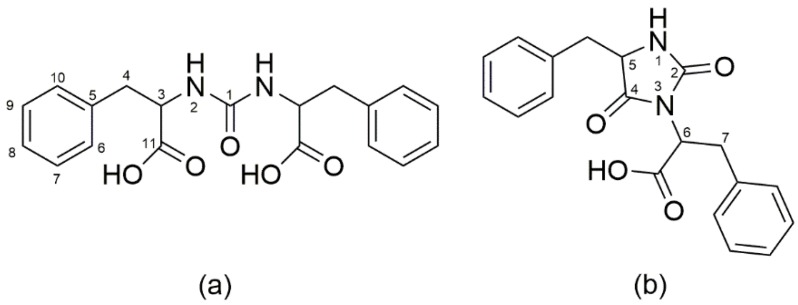
(**a**) Urea derivative of Phe–Phe; (**b**) Hydantoin derivative of Phe–Phe [69].

**Figure 6:**
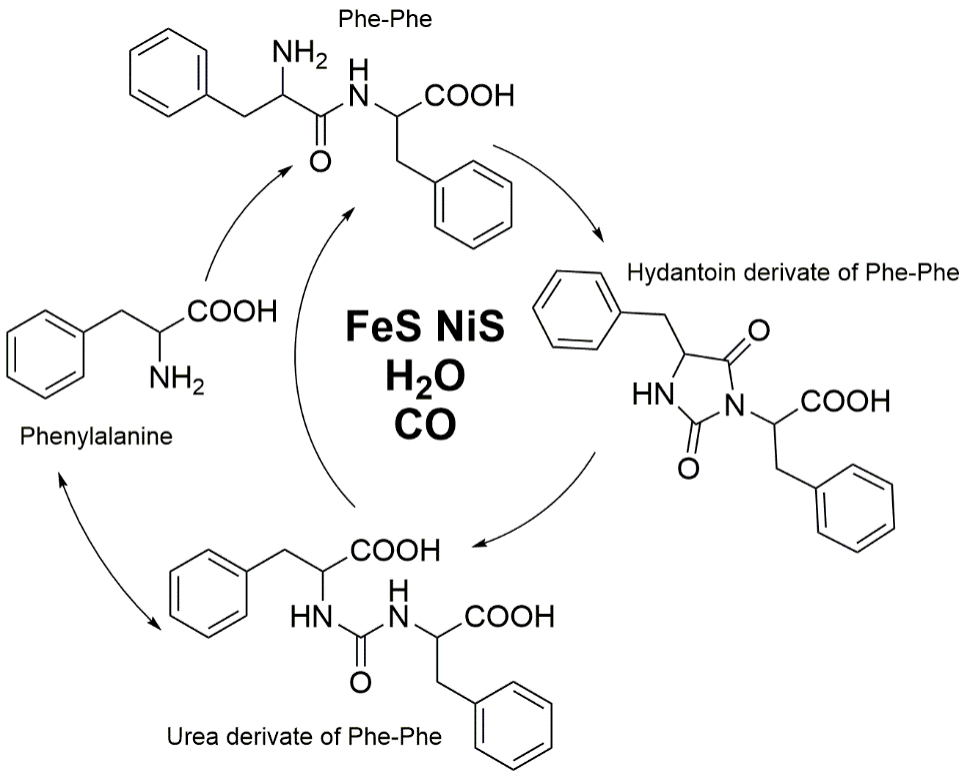
FeS, NiS catalyzed, CO driven peptide cycle as observed for phenylalanine [69].

During LC-MS analysis of the formed peptides (see above), we detected unknown signals in our chromatograms with repeating mass distances to the formed di-, tri-, and tetra-peptides. One class of compounds showed a mass of +44 au whereas a second set showed a mass of +26 au [69]. The mass increment of 44 may suggest an addition of CO_2_, whereas the increment of 26 (= 44 − 18) is probably due to water elimination from these compounds. For structure elucidation of these compounds and also to gain insights into the reaction mechanism, we further took advantage from NMR analysis. Prior to analysis, purification was performed for the M+44 (compound A) and M+26 (compound B) product of Phe–Phe with the help of HPLC on a reversed phase column (C18) and UV detection at 254 nm. The collected fractions were freeze dried and solved in ^2^H_6_-DMSO. ^1^H, ^13^C and two-dimensional (COSY, NOESY, HMQC, and HMBC) NMR studies were performed. Careful signal assignments resulted in the identification of the urea derivative of Phe–Phe as compound A (Figure 5a) and the hydantoin derivative (compound B) of Phe–Phe (Figure 5b). Both compounds were found in two diastereomeric forms. With this structural information and further experiments, a primordial peptide cycle (Figure 6) was postulated [69].

In the “origin-of-life” research, NMR techniques were also used for the detection of phosphorylated compounds such as acetyl phosphate, AMP, ADP, and ATP [70], which are often problematic in mass spectrometric measurements due to sensibility and properties in derivatization. In ^1^H-NMR and ^31^P-NMR spectra, they show characteristic signals which are well resolved and can therefore be analyzed with high sensitivity.

## 5. Stable Isotope Labeling as Advanced Tools to Verify Products and to Elucidate Mechanisms in Product Formation

The products in our reaction settings, e.g., amino acids, hydroxyl acids, and fatty acids, are key molecules in modern and extant life. Highly sensitive analytical tools may therefore produce signals due to contamination through the biological background. For excluding this error possibility as well as for mechanistic insights, stable isotope techniques were highly useful in most of our experiments. Thus, in our settings, reactions were performed in the presence of ^13^CO, ^13^CN^−^, or in D_2_O [39,41,42,45].

**Scheme 1:**
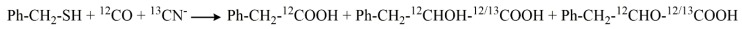
Mixed use of CO and CN- in double carbonylation reactions as shown by stable isotope labelling. Freshly precipitated [Ni(OH)(^13^CN)] or [Co(OH)(^13^CN)] was used as catalyst at pH 12.6 and 60 °C [42].

For example [42], we described carbonylation and double carbonylation of mercaptans with CO and CN^−^, which resulted in corresponding acetic acid, pyruvic acid and lactic acid. For technical reasons, we choose benzyl mercaptane as an educt for most of the reactions. By the use of ^13^CN^−^, we saw the mixed use of carbonyl and cyano groups in these reactions. The carboxyl-C of phenyl acetate, the monocarboxylation product, and the -CH_2_OH-C of phenyl lactate as well as the keto-C of phenyl pyruvate, the double carbonylation products, only derived from CO. For the formation of the carboxyl-C in the double carbonylation products, both CO and CN^−^ were used (Scheme 1).

In further experiments, we studied whether the addition of amino or hydroxy acids, products of many reactions occurring in our system, can influence the yields of the double carbonylation. As described in [42], glycine and alanine promoted double carbonylation up to a factor of five. In addition, we could see a shift in the use of CO and CN^−^ in the carboxyl-C of phenyl lactate. In our standard setting containing 2 mmol NiSO_4_, 2 mmol K^13^CN, 0.5 mmol benzylmercaptan, 1 g Ca(OH)_2_ in 10 mL H_2_O under 60 bar CO pressure at 100 °C for 20 h, we saw 30–35% cyanide-^13^C in the carboxyl group of phenyl lactate. By adding 0.1 mmol glycine, the use of cyanide-C increased to 50–55%. By adding 0.1 mmol lactate, the use of cyanide-C decreased to 10–20%, whereas the yield for the double carbonylation product was not changed (unpublished results). The results showed the possible influence of products from different reactions under volcanic hydrothermal conditions. Products of one reaction act as organic ligands in a different reaction which runs nearby on the same catalytic surface. These results also showed the advantages of stable isotopes for our work, unfortunately sometimes hindered by pricing or availability of the labeled educts.

Other groups also benefited from the advantages of stable isotope labeling to distinguish their products from eventual analytical bias or to get more insight into reaction mechanisms. ^13^C-labeled potassium cyanide was also used by the Sutherland group to follow the formation of sugars via UV radiation [71]. Other ^13^C-labeled precursors like ^13^C-solid carbon or ^13^C-acids were also used to confirm reaction products in the synthesis of fatty acids [45] or other types of acids, like amino and hydroxyl acids [72].

Stable isotope techniques can also be utilized to support the relevance of certain molecules by characterizing the metabolism of phylogenetically ancient organisms. In addition to our chemoautotrophic bottom-up approach, based on chemical reactions under possible primordial conditions, this top-down approach could yield insights into the life style of early organisms and potential fingerprints for a chemoautotrophic “origin-of-life”. For this purpose, especially hyper thermophilic autotrophic organisms are of high interest as they could be the closest descendants from the last universal common ancestor [73]. Reaction pathways and the composition of reactive enzyme centers of these ancient microorganisms can also be used as guidelines in adjusting laboratory experiments.

In an autotrophic “origin-of-life” scenario, carbon-fixation plays a pivotal role. Indeed, stable isotope experiments allowed the identification of several metabolic road maps for “early” carbon fixation [74,75,76]. For example, the discovery of the dicarboxylate/4-hydroxybutyrate pathway in the hyper thermophilic Archaeum *Ignicoccus hospitalis* was based on “feeding” experiments using labeled carbon tracers. Especially NMR allowed for the discovery of the novel reaction cycle [74,77]. The resemblance to the iron-sulfur chemistry in the Wächtershäuser’s theories indicates a possible linkage between the abiotic chemistry in the Hadean Earth and extant biochemistry.

## 6. Extending the “Chemical Space” in “Origin-of-Life” Research by FT-ICR-MS

**Figure 7:**
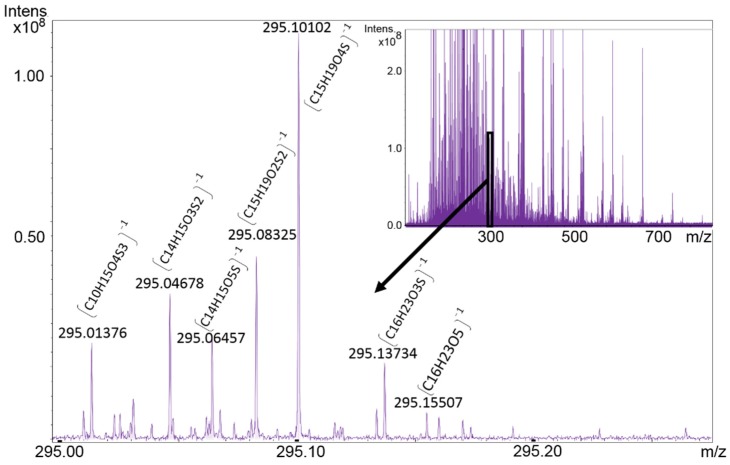
(Inset) FT-ICR-MS spectrum ranging from 120 to 800 m/z of a typical product mixture in our “origin-of-life” research. The spectral region around the nominal mass 295 m/z is displayed showing the high resolution and annotated elemental composition of the negatively charged ions.

Fourier transform ion cyclotron resonance mass spectrometry (FT-ICR-MS) was introduced in the 1970s as new MS technique with surpassing sensitivity and resolution. The mass-to-charge ratio (m/z) of ions is determined based on their cyclotron frequency by passing a detector plate on an orbit. The frequency is direct proportional to the m/z ratio. The resolution of the FT-ICR-MS depends on the magnetic field strength and the measurement time. More detailed information can be found in the contributions of Marshall et al. [78,79]. Recent analysis of the previously described experiments by FT-ICR-MS revealed an unprecedented diversity of products including sulfur containing compounds, previously undetected by GC-MS (Figure 7). Discoveries like this could open up the way for new hypotheses and a better understanding of the underlying reactions taking place [80].

**Figure 8:**
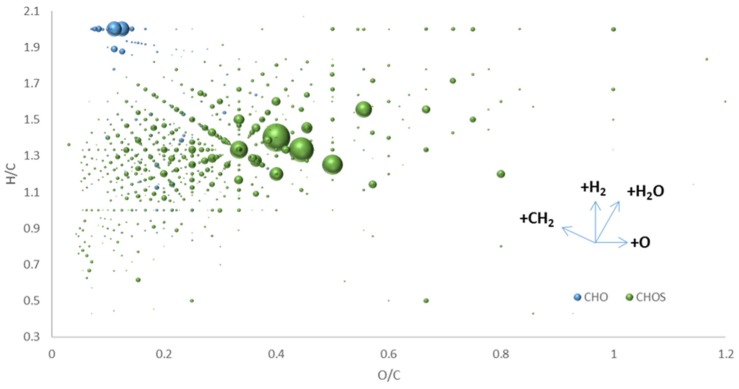
Van Krevelen diagram showing the CHOS chemical space from an experiment performed at 105 °C for 7 days with Ni, CO, and C_2_H_2_, analyzed by FT-ICR-MS.

Visualization of the high amount of different elemental compositions in the analyzed mixtures by Van Krevelen diagrams [81] (plotting of the H/C elemental ratio against the O/C elemental ratio; Figure 8) or other visualization techniques (e.g., Kendrick mass defect [82]) leads to plots that make characteristic structural differences and series of homologous structures easily recognizable between various mixtures.

Introducing FT-ICR-MS measurements has the potential to revolutionize “origin-of-life” research. For example, relevant reaction networks such as Maillard reactions [83] showed unprecedented resolution and widened our view into the reaction mechanisms and kinetics occurring in these reactions. FT-ICR-MS allows to monitor the evolution of multiple compounds at the same time and the reactions between them. Intermediates can be detected and the pathways forming the final products elucidated. Analyzing those reactions as a network and not as single reactions leads to a better understanding of the real metabolic processes. Ongoing FT-ICR-MS data evolution of samples related to the “origin-of-life” will certainly extend the “analytical space” that can be covered and thus, provide a new basis for describing the highly diverse compound mixtures from which metabolism and life could have emerged.

## 7. Discussion

“Origin-of-life” research in general and the chemoautotrophic approach of G. Wächtershäuser in particular is characterized by the attempt to reconstruct the chemical evolution of the Hadean eon. The molecular evolution from very simple molecules which can be found in volcanic hydrothermal exhalations to more complex building blocks of life requires the use of ever more sophisticated analytical tools. Only with the combination of high sensitivity, broad applicability, and structural information accomplished by modern analytical methods the challenges in this field of science can be mastered.

In this review, we emphasize on analytical methods which are used in our own analytical line for detecting small to medium sized organic products in a chemoautotrophic origin of life. These methods can be supplemented by further analytical tools if necessary. For example, capillary electrophoresis was used for analyzing hydrolysis products of Schreibersite [(Fe,Ni)_3_P] [53], IR spectroscopy was used for identifying peptide bonds in wet dry cycles [84] and organic molecules in space [85], X-ray powder diffractometry (XRD) and electron microscopy were used for additional characterization of the inorganic minerals [42].

Despite all improvements in analytical instrumentation, some points may not be disregarded, which may lead to false positive or false negative results. First of all, the purity of all educts and involved chemicals for extraction or elution has to be checked carefully. Even trace compounds may cause false positive results if concentrated or analyzed by highly sensitive methods. Some methods itself are error prone to false positive results e.g., GC-MS columns and injectors show so called memory effects. Due to this problem, a highly concentrated standard or sample injection may cause false positive signals in the following product analysis. Drying procedures like evaporation or freeze drying have also to be checked carefully for possible condensation or polymerization reactions of the molecules under study. This is especially important under the point of view that freeze-thaw [86] and wet-dry cycles [84,87] could have played a role in primordial synthesis of polymers on early Earth. For excluding these possibilities of false positive results, the use of chemicals with the highest purity grade available is essential. Additionally, all chemicals, alone and in mixtures, have to be checked for impurities with the same concentration steps, drying routines and analytical procedures as used later on for the reaction products. GC-MS blank runs have to be checked carefully for possible memory effects and contaminants. Stable isotopes (e.g., ^13^C, ^2^H, ^15^N) should be used as far as possible in the reaction setups for excluding false positive results due to ubiquitous abundant molecules as e.g., amino acids. Furthermore, stable isotope labeling allows the observation of reaction pathways and gives insight into the composition of unknown products. False negatives also have to be taken into account, as the loss of e.g., volatiles or sensitive molecules during sample preparation cannot be compensated by high tech analytical measurements. “Origin-of-life” setups are performed mainly under aqueous conditions to match primordial requirements. Analytical tools like GC-MS or many derivatization methods require the total absence of water. Derivatization methods itself have to be chosen carefully as all of them are restricted to certain reactive groups. This possible loss of reaction products has to be taken into account and additional analytical methods, e.g., LC-MS or NMR, have to be used. Some of these pitfalls are outlined in detail by Keller and Ralser [88]. Additionally, volatile substances could be detected by extraction with organic solvents, head space GC-MS with direct injection of the gaseous phase [89] or solid phase micro extraction (SPME) [90]. LC-MS and NMR allow analysis without derivatization. Especially NMR studies will be prohibited by free metal cations as Fe^2+^ or Ni^2+^, which constitute luckily no problem in our setups with FeS and NiS due to the insolubility of these sulfides. They can be removed easily by centrifugation. If necessary, residual Fe^2+^ or Ni^2+^ could be bound by adding trace amounts of ethylenediaminetetraacetate (EDTA) or related complex builders. If, in different setups, ion exchange procedures are necessary before analysis these have to be tested carefully for possible effects on the products.

Considering all these challenges, it is ever more important to use a complementary set of analytical procedures and tools for elucidating the evolution in “origin-of-life”. An evolving metabolism is characterized by simultaneous anabolic and catabolic reactions forming a network with intermediates in low steady-state concentrations. These can only be handled if sensitive analytical methods are used that can capture multiple reactions or entire pathways at once.

## 8. Working Protocols

### 8.1. Protocol for GC-MS Analysis

In our analytical line, we use a Shimadzu GCMS 2010 plus or ultra, equipped with a fused silica column Equity TM5 and NIST11 as library.

With derivatization:

- Take 1.5 mL reaction mix from reaction vessel; serum flask: syringe/needle; autoclave: pipette
- Centrifuge at 10,000 rpm for 10 min, removing solids from liquid sample
- Fill supernatant in 4 mL vial
- Freeze sample completely at −20 °C
- Freeze dry sample for minimum 24 h to remove all water from sample (derivatization agent and GC/MS column is sensitive to water)
- Dissolve sample in 250 µL Acetonitril (ACN) water free
- Add 250 µL MTBSTFA (*N-tert-*Butyldimetylsilyl-*N-*methyltrifluoroacetamide with 1% *tert*-Butyldimethylchlorosilane)
- Derivatize at 70 °C for 30 min
- Transfer 100 µL to an autosampler vial with 200 µL inlet.
- Measure on GC-MS

Without derivatization:

- Take 1.5 mL reaction mix from reaction vessel
- Centrifuge at 10,000 rpm for 10 min, removing solids from liquid sample
- Separate supernatant and residue
- Extract supernatant/residue with ethyl acetate or *n*-hexane
- Dry solvent supernatant with sodium sulfate (Na_2_SO_4_)
- Transfer 100 µL to an auto sampler vial with 200 µL inlet
- Measure directly with GC-MS without derivatization

Used equipment and conditions:

Shimadzu (Duisburg, Germany) GCMS QP-2010 Plus:

Method 1: 0.2 µL Injection, Injection Temp. 260 °C, Split 1/10; 60 °C for 3 min, with 10 °C/min to 280 °C hold for 3 min; pressure 72.8 Pa; Equity TM5 30 m Film 25 µm; MS Ion source 200 °C, Interface 260 °C, EI, 40–700 m/z, scan speed 1428, column flow 1.20 mL/min; take care of overloaded signals due to derivatization agent
Method 2: 0.5 µL Injection, Injection Temp. 260 °C, Split 1/5; 90 °C for 3 min, with 10 °C/min to 310 °C hold for 10 min; pressure 82.8 Pa; Equity TM5 30 m Film 25 µm; MS Ion source 200 °C, Interface 260 °C, EI, 40–950 m/z, scan speed 2000, column flow 1.17 mL/min; take care of overloaded signals due to derivatization agent

### 8.2. Protocol for LC/MS Analysis

- Take 1.5 mL of reaction mix from reaction vessel
- Centrifuge at 10,000 rpm for 10 min, removing solids from liquid sample
- Take supernatant to 4 mL vial
- Filter 200 µL through 0.45 µm PTFE membrane filter into auto sampler vial with 200 µL inlet
- Measure with HPLC or LC-MS

Used equipment and conditions:

HPLC Merck-Hitachi (Düsseldorf, Germany) Pump L-7100, Merck-Hitachi L-7400 UV-detector
Nucleosil 100-5-C18 5 µm column (Bischoff Chromatography)
A: H_2_O, 1‰ H_3_PO_4_, B: MeOH, 1 ‰ H_3_PO_4_; linear gradient 20–90% B; Flow rate 1 mL/min
Injection: 10 µL
LC-MS Hewlett-Packard (Böblingen, Germany) series 1100 (HPLC), LCQ Finnigan Mat (MS)
Hypersil Gold AQ RP-18 5 µm column
A: H_2_O, B: ACN; linear gradient 10–60% B for 24 min; Flow rate 0.7 mL/min
Ionization: ESI positive mode
Injection: 1 µL

### 8.3. Protocol for NMR Analysis

In general products were dissolved in 500 µL deuterated solvents. ^1^H and ^13^C spectra were recorded at 500.13 MHz and 125.76 MHz, respectively, using a Bruker DRX500 or an AVANCE 500 spectrometer equipped with a cryo probe head. Two dimensional COSY, NOESY, HMQC, and HMBC experiments were measured with standard Bruker parameters (XWINNMR 3.0).

In D_2_O:

- Take 1.5 mL reaction mix from reaction vessel
- Centrifuge at 10,000 rpm for 10 min, removing solids from liquid sample
- Transfer supernatant to 4 mL vial
- Freeze sample completely at −20 °C
- Freeze dry sample for minimum 24 h to remove extant water from sample
- Dissolve dried residue in 520 µL D_2_O
- Add 40 µL of TSP (sodium trimethylsilyl propionate) as internal standard to calibrate 0.0 ppm
- Transfer sample to NMR tube

In other solvents:

- Take 1.5 mL reaction mix from reaction vessel
- Centrifuge at 10,000 rpm for 10 min, removing solids from liquid sample
- Separate supernatant and residue
- Extract supernatant/residue with e.g., 520 µL of CDCl_3_ or d_6_-benzene
- Dry solvent with sodium sulfate (Na_2_SO_4_)
- Transfer solvent phase in NMR tube
- Add 40 µL of TMS (tetramethylsilane) to calibrate 0.0 ppm

### 8.4. Protocol for FT-ICR/MS and Data Analysis

- Take 100 µL reaction mix from reaction vessel
- Centrifuge at 15,000 rpm for 5 min, removing solids from liquid sample
- Take 6 µL supernatant
- Dilute in 994 µL Methanol
- Direct infusion at 2 µL/min
- Formula assignment following Tziotis et al. [91]

## Supplementary Materials

Supplementary File 1
