# Supplementary figures and images for: Evolutionary Steps in the Analytics of Primordial Metabolic Evolution
Source: Life (Basel). 2019 Jun 18;9(2):50. doi: 10.3390/life9020050 (PMC6616974; doi:10.3390/life9020050)

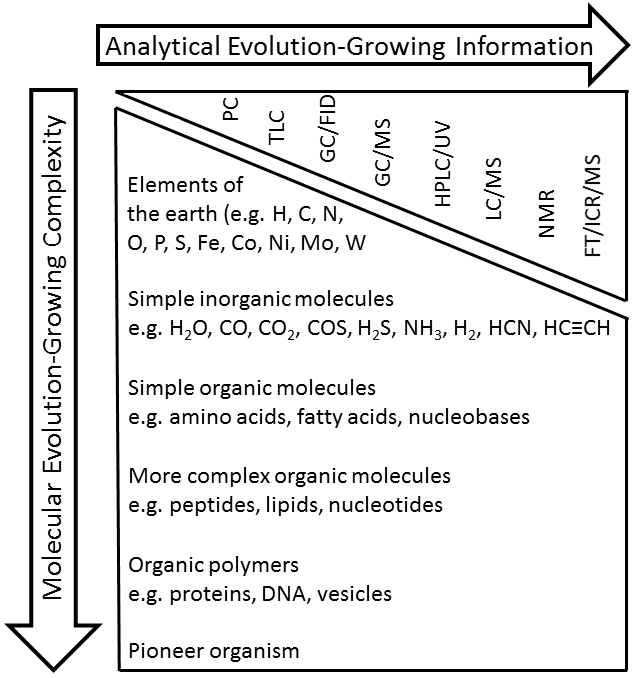

Supplement: Supplementary File 1 [file life-09-00050-s001.zip › Figures_Geisberger/Figure 1.tif]

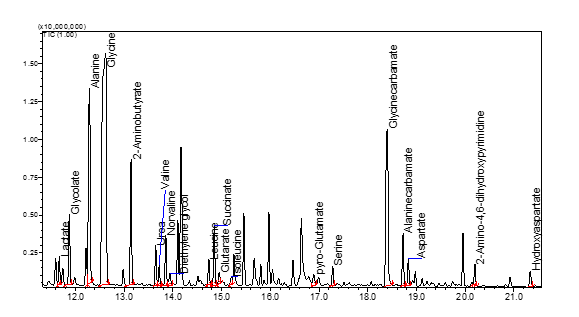

Supplement: Supplementary File 1 [file life-09-00050-s001.zip › Figures_Geisberger/Figure 3.tif]

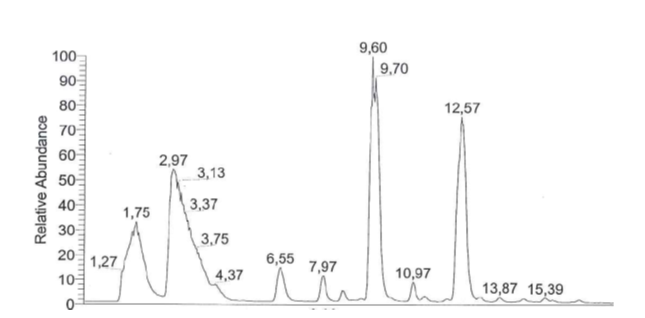

Supplement: Supplementary File 1 [file life-09-00050-s001.zip › Figures_Geisberger/Figure 4.tif]

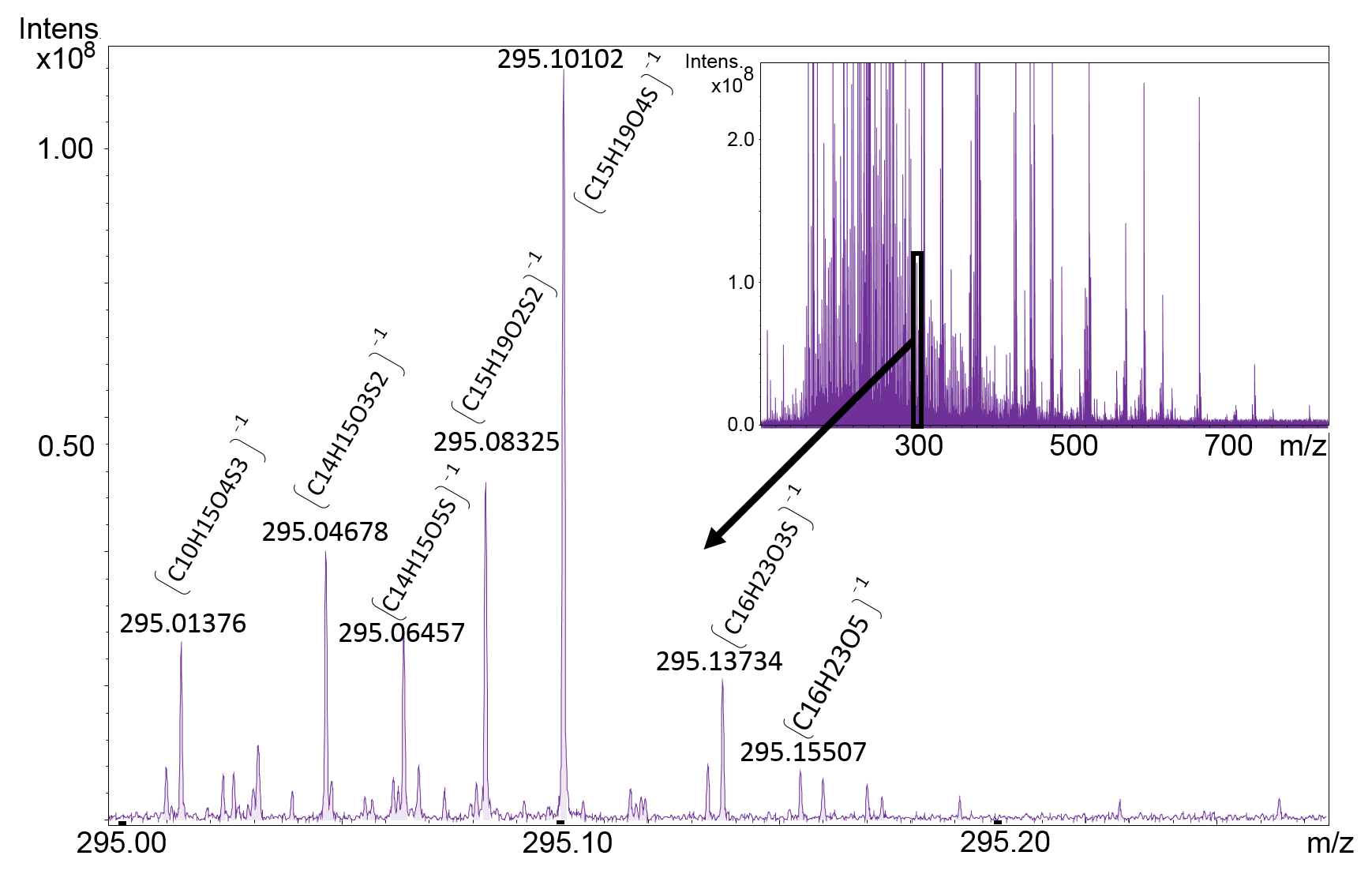

Supplement: Supplementary File 1 [file life-09-00050-s001.zip › Figures_Geisberger/Figure 7.tif]

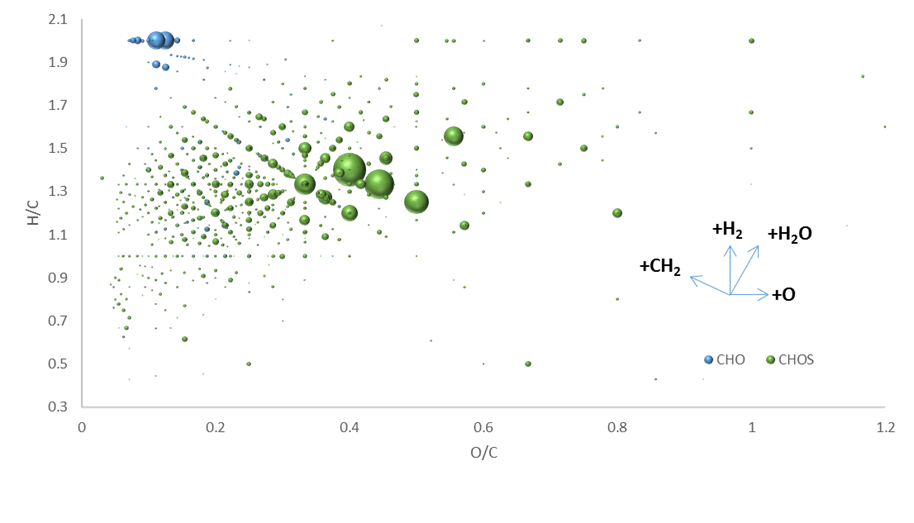

Supplement: Supplementary File 1 [file life-09-00050-s001.zip › Figures_Geisberger/Figure 8.tif]
